# Comparative Analysis of Cardiometabolic Risk Profiles Among Hypertensive and Normotensive Adults: Cross-Sectional Study

**DOI:** 10.2196/84466

**Published:** 2026-09-10

**Authors:** Lebogang Faith Thaga, Gudani Goodman Mukoma, Terry Jeremy Ellapen, Takalani Clearance Muluvhu

**Affiliations:** 1 Biokinetics, Recreation and Sport Science Health Science University of Venda Thohoyandou, Limpopo South Africa; 2 Department of Human Movement and Therapeutic Sciences Faculty of Science Tshwane University of Technology Pretoria, Gauteng South Africa

**Keywords:** adults, cardiometabolic risk, comparative analysis, hypertensive, South Africa, obesity

## Abstract

**Background:**

Hypertension is a significant modifiable risk factor for cardiometabolic syndrome, leading to increased morbidity and premature mortality. Understanding the differences in cardiometabolic risk profiles between hypertensive and normotensive adults is essential for effective prevention strategies.

**Objective:**

This study examined cardiometabolic risk profiles among adults in Johannesburg South, Gauteng Province, South Africa.

**Methods:**

A cross-sectional study was conducted with 209 adults aged 25 to 65 years who were recruited from 3 Johannesburg townships in Region G. Anthropometric measures (BMI and waist circumference), blood pressure, and biochemical indexes (random glucose, total cholesterol, high-density lipoprotein cholesterol, low-density lipoprotein cholesterol, and triglycerides) were assessed. Statistical analyses were performed using Stata (version 19), with statistical significance set at *P*<.05.

**Results:**

Hypertension prevalence increased significantly with BMI (*P*<.001) and waist circumference (*P*=.003). In multivariable analysis, age and elevated glucose levels were independently associated with hypertension, whereas obesity and central adiposity were not significantly associated after adjustment.

**Conclusions:**

Hypertension was independently associated with advancing age and elevated glucose levels in this population. Although obesity and central adiposity showed significant associations in unadjusted analyses, they were not independently associated with hypertension after adjustment. These findings highlight the importance of early screening and targeted interventions to address cardiometabolic risk in underserved South African communities.

## Introduction

Hypertension remains a persistent global health challenge, significantly elevating the risk of cardiovascular and other noncommunicable diseases (NCDs). These conditions often lead to long-term disability. In 2017, NCDs accounted for 67% of all-age disability-adjusted life years in sub-Saharan Africa [1]. Commonly referred to as a silent killer, hypertension typically presents no obvious symptoms, making early detection difficult [2]. Regular screening is a reliable diagnostic method; however, it remains largely inaccessible in many low- and middle-income countries, including South Africa. As a result, the prevalence of hypertension continues to rise, particularly in countries in the World Health Organization (WHO) African region, where it stands at 27%, notably higher than the 18% reported in the WHO American region [3].

With the rising prevalence of hypertension, research has increasingly focused on cardiometabolic risks, highlighting obesity as the most prominent predictor [4,5]. Onagbiye et al [6] identified abdominal obesity as a major contributor to cardiometabolic disease among university staff employees. Similarly, Modjadji et al [4] found that the odds of developing hypertension were high for obese truck drivers based on waist circumference (WC; adjusted odds ratio [AOR] 4.68, CI 1.92-11.34) and waist-to-height ratio (AOR 5.49, CI 1.74-17.27). Interestingly, a study among a younger population also concurred with these findings [7]. Despite consistent evidence across different populations, regions, and demographic groups, the global prevalence of overweight and obesity continues to increase.

It is important to note that a decade ago, central obesity was proven to be the key determinant of the prevalence of metabolic syndrome in sub-Saharan Africa [8]. Efforts to address this burden have encountered several challenges. For instance, a recent study in Kenya revealed a high prevalence of central obesity, with women being significantly more affected than men (58.3% compared to 35.6%) [9]. Furthermore, the 2025 WHO report highlights a continuing upward trend in global obesity rates, noting that 43% of adults aged ≥18 years are overweight and 16% are living with obesity. This growing trend is concerning, as obesity significantly increases the risk of developing type 2 diabetes mellitus, hyperlipidemia, hypertension, and other cardiovascular diseases [10]. These illnesses often coexist, increasing health risks and reducing life expectancy. Their combined effects extend beyond individuals; they have a substantial negative impact on the national economy, health care systems, and workplace productivity.

The increased cost of treating associated comorbid diseases related to obesity imposes a substantial financial burden on the South African health care system. In particular, overweight and obesity accounted for ZAR 28,734 million (ZAR 1=US $0.060 as of July 29, 2026) in expenses related to cardiovascular and endocrine diseases, with approximately 91% of this cost coming from hypertension and diabetes [11]. These figures are alarming, as access to quality health care varies widely across South Africa. Currently, 44% of all health care expenditure occurs in the private sector, serving only 16% of the South African population [12]. This means the rest of the population relies on a public health system that is underresourced and often inaccessible due to geographical, financial, or knowledge barriers [13].

Given this background, comprehensive profiling of cardiometabolic risk factors in underresourced communities is essential to inform targeted prevention and early intervention strategies. Although previous South African studies have demonstrated associations between obesity, central adiposity, and hypertension, most have focused on occupational cohorts, such as truck drivers and university staff, with limited evidence from community-dwelling adults living in underresourced urban settings. Consequently, there remains a significant gap in localized epidemiological data describing cardiometabolic risk profiles among township populations. This study addresses this gap by examining the cardiometabolic risk profiles of adults residing in selected townships in Johannesburg South. Specifically, the study aimed to compare the cardiometabolic risk profiles of hypertensive and normotensive adults and to identify independent predictors of hypertension.

## Methods

### Study Design and Participants

**Figure 1:**
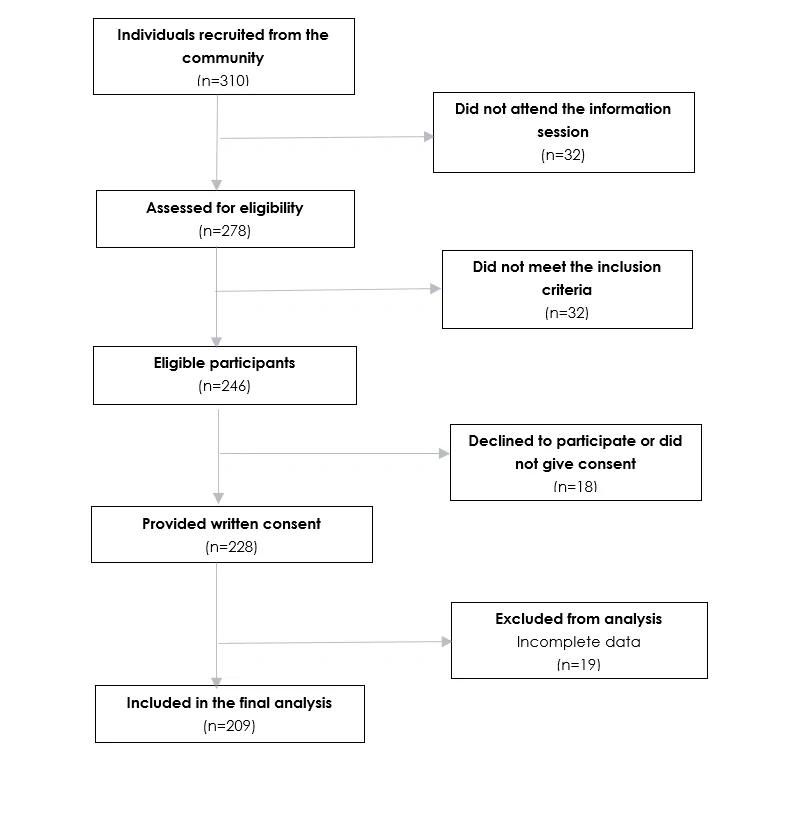
Flow diagram of participant recruitment, eligibility assessment, enrollment, exclusion, and inclusion in the final analysis.

This cross-sectional study used purposive sampling to recruit male and female adults aged 25 to 65 years from selected townships in Johannesburg South. Although purposive sampling may limit representativeness and external validity, it was the most feasible approach because of the absence of reliable sampling frames in informal township settings. This approach enabled the inclusion of underrepresented community members and provided valuable baseline evidence to inform future probabilistic studies. A total of 310 individuals were recruited. Of these, 101 (32.6%) were excluded because they did not attend the information session (n=32, 10.3%), did not meet the inclusion criteria (n=32, 10.3%), declined to provide informed consent (n=18, 5.8%), or had incomplete anthropometric or biochemical data (n=19, 6.1%). The final analytic sample comprised 209 participants (n=72, 34.4% men and n=137, 65.6% women) with complete data for all study variables, including demographic characteristics, blood pressure, anthropometric measurements, and biochemical indexes. Missing data were handled using listwise deletion; therefore, no imputation methods were applied. Analyses were restricted to complete cases to maintain the consistency and reliability of statistical comparisons. Participant recruitment, eligibility assessment, enrollment, exclusion, and inclusion in the final analysis are summarized in Figure 1.

Between 2023 and 2025, participants were recruited from community churches, households, and social gatherings in selected townships within Region G of the city of Johannesburg using a purposive sampling approach. Region G is the second most populated administrative region in the municipality and experiences considerable socioeconomic deprivation [14]. Eligible participants were permanent residents of the 3 selected townships and were aged 25 to 65 years.

The city of Johannesburg covers approximately 1645 km^2^ in Gauteng Province. The central and northern suburbs are predominantly occupied by middle- and upper-income populations, whereas the southern suburbs and the far northern fringes are characterized by greater socioeconomic disadvantage. Approximately 20% of the city of Johannesburg population lives in informal settlements with limited access to basic municipal services, while a further 40% resides in inadequate housing with insufficient municipal support [15].

### Ethical Considerations

This study forms part of a PhD project approved by the Faculty Committee for Research Ethics–Science (FCRE-SCI), Tshwane University of Technology (FCRE-SCI 2023/05/016 [SCI; FCPS 02]). The study was conducted in accordance with the ethical principles of the Declaration of Helsinki and its subsequent amendments. Written informed consent was obtained from all participants prior to enrollment. Participants with elevated blood pressure, abnormal blood glucose levels, or other clinically significant findings identified during the screening were informed of their results and verbally advised to visit their nearest primary health care facility for further clinical evaluation and management.

### Instrumentation

#### Anthropometric Measurements

Anthropometric measurements were taken after participants completed the questionnaires, with assistance from research assistants or the principal investigator. Body mass was recorded to the nearest 0.1 kg, using a calibrated BFW 300 Platform Scale (Adam Equipment Co Ltd), and body stature to the nearest 0.1 cm, using the portable Seca 213 stadiometer (CE 0123) according to the validated protocols of the International Society for the Advancement of Kinanthropometry [16]. BMI was categorized according to the American College of Sports Medicine cut points [17] as follows: BMI<18.5 kg/m^2^=underweight, BMI 18.5-24.9 kg/m^2^=normal, BMI 25.0-29.9 kg/m^2^=overweight, and BMI≥30.0 kg/m^2^=obese.

WC measurements were recorded to the nearest 0.1 cm using a nonstretchable standard Lufkin tape measure manufactured by Cooper Tools of Apex. The WC risk measurements were categorized according to low risk (WC<80 cm in women and <94 cm in men) and high risk (WC≥88 cm in women and ≥94 cm in men) [18].

#### Biochemical Measurements

Blood pressure was measured with the arm supported at the heart level (European Society of Hypertension) with a sphygmomanometer (Omron) using the Riva-Rocci or Korotkoff method on the left arm [19]. An appropriately sized blood pressure cuff was used, and participants were instructed to refrain from smoking cigarettes and from ingesting caffeine for 30 minutes prior to measurements. Measurements were taken thrice with a 5-minute resting period between each measurement [20]. The average of the measurements was used for data analysis.

The blood pressure cutoff points used for analysis were assessed according to guidelines of the American College of Cardiology and American Heart Association, where normal blood pressure is defined as <120 mm Hg systolic and <80 mm Hg diastolic, elevated blood pressure as 120 to 129 mm Hg systolic and <80 mm Hg diastolic, stage 1 hypertension as 130 to 139 mm Hg systolic or 80 to 89 mm Hg diastolic, and stage 2 hypertension as ≥140 mm Hg systolic or ≥90 mm Hg diastolic [19]. Participants who were on hypertensive medication with a known diagnosis were considered hypertensive. Participants who presented with hypertension at baseline and who were not on medication were referred to the clinic.

A peripheral blood sample was collected to determine random glucose levels, total cholesterol, high-density lipoprotein cholesterol (HDL-C), low-density lipoprotein cholesterol (LDL-C), and triglycerides. The values were recorded in millimoles per liter according to the manufacturer’s CardioChek Plus analyzer user guide v1.12 or later. Random glucose levels <7.8 mmol/L were classified as low risk and ≥7.8 mmol/L as high risk. Random glucose was a practical measure in township settings where fasting requirements and laboratory access for hemoglobin A_1c_ (HbA_1c_) testing were not feasible, whereas less precise, random glucose testing would have allowed immediate, point-of-care screening and referral, thereby ensuring participation from underresourced communities. Cholesterol norms were classified as total cholesterol<5 mmol/L=normal, LDL-C<3 mmol/L=normal, HDL-C<1.04-mmol/L=low, and HDL-C≥1.2 mmol/L=optimal. Triglyceride concentrations were classified as normal (<1.7 mmol/L) and high risk (≥1.7 mmol/L) [18]. Persons taking antiglycemic medication were also classified as diabetic.

### Statistical Analysis

Data were cleaned, coded, and analyzed using Stata SE (version 19; StataCorp). The distribution of continuous variables was assessed using the Shapiro-Wilk test, histograms, and Q-Q plots to evaluate normality and guide the selection of appropriate descriptive statistics. As the continuous variables were not normally distributed, they are presented as medians with IQR. Categorical variables are presented as frequencies and percentages. The prevalence of cardiometabolic risk factors and hypertension was summarized using proportions.

A multivariable logistic regression (LR) model was used to identify independent predictors of hypertension. Prior to model fitting, the assumptions of LR were evaluated. The outcome variable was binary (hypertensive vs normotensive), observations were independent, multicollinearity was assessed using variance inflation factors, and model fit was evaluated using the Hosmer-Lemeshow goodness-of-fit test. Variance inflation factor values ranged from 1.11 to 1.93, indicating no evidence of problematic multicollinearity among the predictors included in the model. Statistical significance was set at *P*<.05.

## Results

### Descriptive Statistics

The baseline characteristics of the 209 study participants are summarized in Table 1. The median age was 45 (IQR 32-57) years. Additional anthropometric and clinical characteristics included a median BMI of 27.6 (IQR 15.8-52.7) kg/m^2^ and a median systolic blood pressure (SBP) of 122 (IQR 65-181) mm Hg.

**Table 1:** Demographic and anthropometric characteristics (N=209).

Variables	Median (IQR)
Age (years)	45 (32-57)
BMI (kg/m^2^)	27.6 (24.1-33)
Waist circumference (cm)	94 (82-106)
Systolic blood pressure (mm Hg)	122 (112-134)
Diastolic blood pressure (mm Hg)	80 (71-89)
Glucose (mmol/L)	5.8 (5.2-7)
Total cholesterol (mmol/L)	3.76 (3.18-4.46)
HDL^a^ cholesterol (mmol/L)	1.11 (0.91-1.37)
LDL^b^ cholesterol (mmol/L)	1.97 (1.11-2.48)
Triglycerides (mmol/L)	1.22 (0.8-1.76)

^a^HDL: high-density lipoprotein.

^b^LDL: low-density lipoprotein.

**Figure 2:**
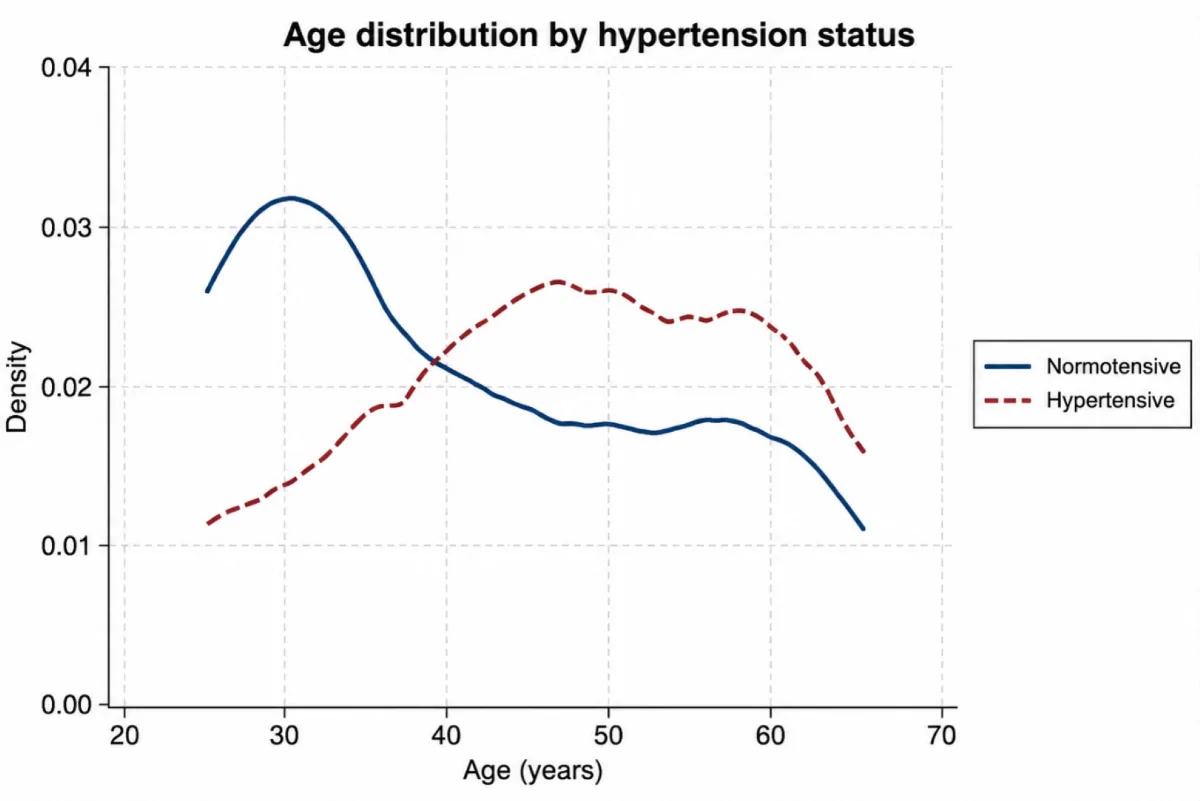
Density plot showing the age distribution of normotensive and hypertensive participants. The blue curve represents normotensive participants, and the red curve represents hypertensive participants.

Figure 2 presents the age distribution of participants according to hypertension status. The normotensive group, represented by the blue curve, peaked in the late 20s to early 30s and gradually declined with increasing age. In contrast, the hypertensive group (red curve) showed increasing density with advancing age, peaking in the late 50s to early 60s. The 2 distributions intersected at approximately 45 to 50 years, suggesting a shift in the age range at which hypertension became more prevalent than normotension. These findings support the well-established association between advancing age and increased hypertension prevalence.

### Association Between Obesity, Central Obesity, and Hypertension Status

Table 2 presents the association between BMI, WC, and hypertension status, which was analyzed using chi-square tests. A statistically significant association was observed between BMI category and hypertension (*P*<.001). The prevalence of hypertension increased progressively with higher BMI, rising from 30.4% (17/56) among individuals with normal BMI to 64.6% (53/82) among those classified as obese. Similarly, WC showed a significant association with hypertension (*P*=.003), with 53.4% (79/148) of participants with high WC being hypertensive, compared to 31.1% (19/61) of those with normal WC.

**Table 2:** Association between obesity, central obesity, and hypertension status among study participants (N=209).

Variable and category		Normotensive, n (%)	Hypertensive, n (%)	*P* value^a^	
**BMI**					<.001
	Underweight	4 (50)	4 (50)		
	Normal weight	39 (69.6)	17 (30.4)		
	Overweight	39 (61.9)	24 (38.1)		
	Obese	29 (35.4)	53 (64.6)		
**Waist circumference**					.003
	Normal	42 (68.9)	19 (31.1)		
	High	69 (46.6)	79 (53.4)		

^a^*P* values were calculated using Pearson chi-square test.

### Lipid Profiles

The association between several lipid categories and the presence of hypertension is presented in Table 3. Chi-square test results for these variables indicate no statistically significant association with hypertension. The *P* values for HDL cholesterol (*P*=.65), LDL cholesterol (*P*=.70), and triglycerides (*P*=.25) are well above the conventional significance level of .05. This means that, in this dataset, the distribution of hypertension across the categories for these lipid variables is not different from what would be expected by chance.

**Table 3:** Comparison of lipid profile categories between normotensive and hypertensive participants (N=209).

Variable and category		Normotensive (n=111), n (%)	Hypertensive (n=98), n (%)	Chi-square (*df*)		*P* value^a^	
**HDL^b^ cholesterol**					0.2 (1)		.65
	Normal	43 (51.2)	41 (48.8)				
	High risk	68 (54.4)	57 (45.6)				
**LDL^c^ cholesterol**					0.1 (1)		.70
	Normal	99 (52.7)	89 (47.3)				
	High risk	12 (57.1)	9 (42.9)				
**Triglycerides**					1.3 (1)		.25
	Normal	87 (55.4)	70 (44.6)				
	High risk	24 (46.2)	28 (53.8)				
**Total cholesterol**					3.3 (1)		.07
	Normal	102 (55.4)	82 (44.6)				
	High risk	9 (36)	16 (64)				

^a^*P* values were calculated using Pearson chi-square test.

^b^HDL: high-density lipoprotein.

^c^LDL: low-density lipoprotein.

Interestingly, the results for total cholesterol show a borderline association with hypertension. While the *P* value of .07 is slightly above the .05 significance threshold, the association was not statistically significant. The table shows that a lower percentage of participants in the high-risk total cholesterol category (9/25, 36%) had no hypertension, and a higher rate (16/25, 64%) had hypertension, compared with participants in the normal total cholesterol category. This suggests that having a higher total cholesterol level might be associated with an increased likelihood of hypertension, but the finding is not statistically significant at the 5% level.

### Predictors of Hypertension

A multivariable LR model was fitted to identify predictors of hypertension status. The model was statistically significant (LR: *χ*^2^_11_=76.3; *P*<.001), indicating that the predictors collectively explained variation in hypertension. The LR model demonstrated adequate fit to the data based on the Hosmer-Lemeshow goodness-of-fit test (*χ*^2^_8_=7.2; *P*=.52).

As shown in Table 4, age and elevated glucose levels were independently associated with hypertension. Increasing age was positively associated with hypertension (β=0.07, 95% CI 0.041-0.099; *P*<.001). Elevated glucose levels were also independently associated with hypertension (β=2.277, 95% CI 0.684-3.871; *P*<.005). Other variables, including gender, BMI category, WC, and lipid levels, were not significantly associated with hypertension after adjustment. The crude associations observed for BMI and WC in the bivariate analyses may reflect their correlation with advancing age.

**Table 4:** Multivariable logistic regression (LR) analysis of independent predictors of hypertension.

Variable				β (95% CI)		*P* value
**Individual-level factors**						
	Age (years)		0.07 (0.041 to 0.099)		<.001	
	Sex (female vs male)		0.155 (−0.677 to 0.987)		.71	
**Metabolic factors**						
	**BMI category**					
		Normal weight	−1.51 (−3.207 to 0.186)		.08	
		Overweight	−1.238 (−3.031 to 0.556)		.18	
		Obese	−0.058 (−1.874 to 1.757)		.95	
	Waist circumference (high vs normal)		0.124 (−0.966 to 1.214)		.82	
	HDL^b^ cholesterol (high vs normal)		−0.219 (−0.986 to 0.549)		.58	
	LDL^c^ cholesterol (high vs normal)		−1.043 (−2.286 to 0.200)		.10	
	Triglycerides (high vs normal)		−0.178 (−0.984 to 0.628)		.67	
	Total cholesterol (high vs normal)		0.806 (−0.518 to 2.130)		.23	
	Glucose category (high vs normal)		2.277 (0.684 to 3.871)		.005	

^b^HDL: high-density lipoprotein.

^c^LDL: low-density lipoprotein.

Statistical significance was set at *P*<.05. Model fit: likelihood ratio *χ*^2^_11_=76.3.

### Association Between SBP, BMI, and Hypertension

**Figure 3:**
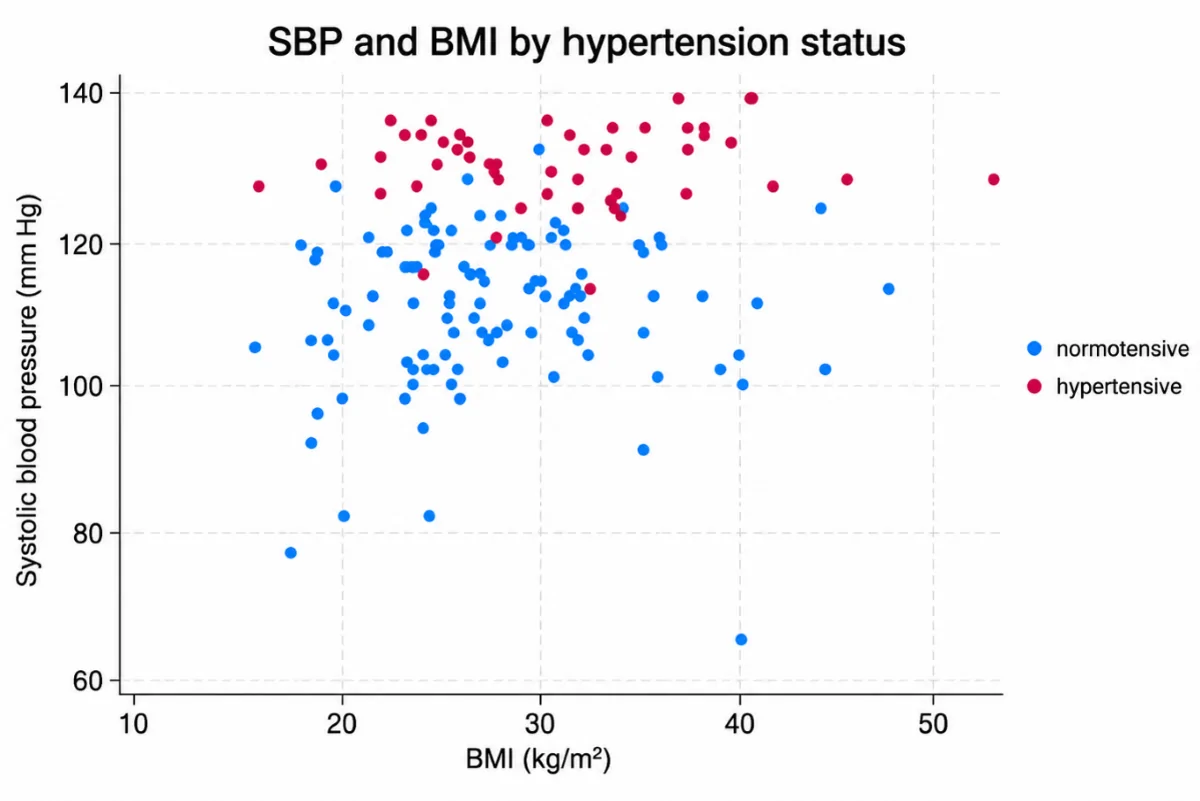
Scatter plot showing the relationship between systolic blood pressure (SBP) and BMI among normotensive and hypertensive participants. Blue points represent normotensive participants, and red points represent hypertensive participants.

Figure 3 illustrates the relationship between SBP and BMI according to hypertension status. Hypertensive participants consistently exhibited higher SBP values than normotensive participants across the BMI spectrum. Although higher BMI values were observed in both groups, substantial overlap was evident, suggesting that BMI alone did not clearly distinguish hypertension status in this population.

## Discussion

### Principal Findings

This study compared cardiometabolic risks between hypertensive and normotensive adults in selected townships of Johannesburg. The primary finding of this study was that older age and elevated glucose levels were the strongest independent predictors of hypertension in the adjusted models. Our analysis showed that increasing age and elevated glucose levels were both independently associated with hypertension. These findings align with established evidence linking advancing age to vascular stiffening and other physiological changes that contribute to elevated blood pressure [21,22].

Although obesity and central adiposity showed significant associations with hypertension in crude analyses, these associations were attenuated primarily in adjusted models. This suggests that the relationship between obesity and hypertension in this population is primarily mediated by age, which plays a dominant role. Consistent with longitudinal evidence, the risk of developing hypertension increases steadily with advancing age [23]. At the same time, cohort studies consistently demonstrate that elevated BMI and WC are strongly linked to hypertension risk across all stages of life, underscoring the critical role of excess adiposity in disease development [23,24].

Notably, life stage–specific research has shown that high baseline BMI is the strongest predictor of hypertension in early adulthood, whereas high WC exerts the greatest impact during childhood [25]. The high prevalence of obesity among hypertensive individuals in our study (53/82, 64.6%) is clinically significant and mirrors global trends [26-28]. While other studies emphasize obesity as a key independent risk factor [29-31], our findings highlight the need to account for the strong independent effect of age when designing targeted interventions for this community.

From a public health perspective, these results suggest that interventions should focus on 2 key areas: addressing excess body fat across the lifespan and tailoring strategies to the specific risk profiles of different age groups. For younger populations, prevention efforts should prioritize weight management, physical activity, and healthy dietary habits to help prevent the onset of hypertension. In contrast, for older adults, interventions should focus on regular blood pressure screening, management of existing health conditions, and lifestyle modifications that are practical and achievable in later life. Community-based programs that combine age-sensitive approaches with obesity prevention strategies could have the greatest impact.

In contrast to other studies that reported dyslipidemia as a prevalent comorbidity [32-34], we did not find statistically significant independent associations between lipid markers (HDL, LDL, and triglycerides) and hypertension. However, the *P* value for total cholesterol was close to the conventional significance threshold (*P*=.07), suggesting a potential trend warranting further investigation. Although these lipid parameters did not reach statistical significance, their prevalence in the population remains clinically relevant and should be considered in risk reduction efforts.

Evidence from larger cross-sectional studies has consistently demonstrated a strong relationship between hypertension and dyslipidemia. For example, a study conducted in Poland reported a significant association between hypertension and abnormal cholesterol levels (*P*<.001), with hypertensive individuals showing a 3-fold increase in the odds of having abnormal cholesterol levels [35]. Similar trends have been observed in sub-Saharan Africa, where dyslipidemia frequently coexists with hypertension and contributes to elevated cardiometabolic risk [33,36]. In light of these findings, the absence of statistically significant associations in our study may be due to sample size limitations, population-specific characteristics, or unmeasured confounding factors, such as diet, physical activity, and socioeconomic status.

The South African context magnifies the importance of these findings. Research from other local populations has demonstrated strong correlations between BMI, WC, and SBP [6,37], mirroring the current study’s outcomes. International evidence further highlights the growing burden of obesity, particularly among middle-aged adults and rural populations [9,28,30]. Together, these results emphasize the urgency of community-level interventions that combine regular health screenings, obesity management, and culturally tailored health education.

Overall, the findings suggest a clustering of cardiometabolic risk factors, with obesity and glucose dysregulation playing central roles in hypertension pathogenesis. The substantial proportion of undiagnosed or uncontrolled hypertension observed in Johannesburg South underscores the need for early detection, integrated screening, and multifactorial interventions.

### Limitations

Several limitations must be acknowledged. First, the cross-sectional design precludes the determination of causal relationships. The observed relationships should be interpreted as correlations rather than causal pathways. Longitudinal studies are necessary to establish temporal and causal links between age, glucose dysregulation, obesity, and hypertension.

Second, although purposive sampling limits representativeness and external validity, it was considered the most feasible approach because of the absence of reliable sampling frames in township settings. This method ensured the inclusion of underrepresented community members, providing valuable baseline evidence to inform future probabilistic studies.

Third, the final sample of 209 participants may have limited statistical power to detect modest associations, particularly for lipid-related variables and within the multivariable LR model. Consequently, some nonsignificant findings may reflect insufficient statistical power rather than the absence of a true association. Larger studies are needed to confirm these findings and improve the precision of the estimates. Furthermore, excluding participants with incomplete data or those who did not return for screening may have introduced selection bias, potentially limiting the representativeness of the final analytic sample. In addition, the underrepresentation of men, which is common in South African epidemiological research, may limit the generalizability of the findings.

Additionally, glycemic status was assessed using random blood glucose measurements rather than fasting plasma glucose or HbA_1c_. Although random glucose testing was the most feasible approach in this community-based setting, it is less precise than standard diagnostic measures and may have resulted in misclassification of glycemic status.

Finally, the study did not include socioeconomic and behavioral factors, such as dietary intake, physical activity, and stress, which may have influenced the observed associations.

### Recommendations

Future research should use longitudinal designs with larger, more diverse samples to strengthen causal inference and generalizability. Incorporating dietary, lifestyle, and socioeconomic data will provide a more comprehensive understanding of modifiable risk factors. Moreover, the use of sensitive lipid biomarkers is essential to elucidate their role in hypertension. Evaluating community-based screening and health education programs could further inform effective prevention strategies.

### Conclusions

Hypertension was independently associated with advancing age and elevated glucose levels in this population. Although obesity and central adiposity showed significant associations in unadjusted analyses, they were not independently associated with hypertension after adjustment. While lipid markers were not statistically significant predictors, their prevalence remains clinically relevant and warrants continued monitoring. These findings highlight the importance of early screening and multifactorial interventions to address cardiometabolic risk in underserved South African communities.

## Abbreviations

AOR: adjusted odds ratio
HbA1c: hemoglobin A1c
HDL-C: high-density lipoprotein cholesterol
LDL-C: low-density lipoprotein cholesterol
LR: logistic regression
NCD: noncommunicable disease
SBP: systolic blood pressure
WC: waist circumference
WHO: World Health Organization
